# COVID-19 and the Response to Antiplatelet Therapy

**DOI:** 10.3390/jcm12052038

**Published:** 2023-03-04

**Authors:** Tomáš Bolek, Matej Samoš, Jakub Jurica, Lucia Stančiaková, Martin Jozef Péč, Ingrid Škorňová, Peter Galajda, Ján Staško, Marián Mokáň, Peter Kubisz

**Affiliations:** 1 Department of Internal Medicine I, Jessenius Faculty of Medicine in Martin, Comenius University in Bratislava, 036 59 Martin, Slovakia; 2 National Centre of Hemostasis and Thrombosis, Department of Hematology and Blood, Transfusion, Jessenius Faculty of Medicine in Martin, Comenius University in Bratislava, 036 59 Martin, Slovakia

**Keywords:** antiplatelet therapy, aspirin, arterial thrombosis, COVID-19

## Abstract

The coronavirus SARS-CoV2 disease (COVID-19) is connected with significant morbidity and mortality (3.4%), disorders in hemostasis, including coagulopathy, activation of platelets, vascular injury, and changes in fibrinolysis, which may be responsible for an increased risk of thromboembolism. Many studies demonstrated relatively high rates of venous and arterial thrombosis related to COVID-19. The incidence of arterial thrombosis in severe/critically ill intensive care unit–admitted COVID-19 patients appears to be around 1%. There are several ways for the activation of platelets and coagulation that may lead to the formation of thrombi, so it is challenging to make a decision about optimal antithrombotic strategy in patients with COVID-19. This article reviews the current knowledge about the role of antiplatelet therapy in patients with COVID-19.

## 1. Introduction

The coronavirus SARS-CoV2 disease (COVID-19) is known to be associated with significant morbidity and mortality. Globally, there are approximately 616 million COVID-19 cases reported to date, with a mortality of 3.4%. Studies have highlighted an astonishing rate of venous thromboembolism (VTE) and pulmonary embolism (PE) in patients with a severe form of COVID-19 reaching 42% and 17%, respectively [1]. However, arterial thrombotic events have also been described at various sites, such as coronary arteries, cerebral arteries, and peripheral arteries [2,3]. A pharmacologic thromboprophylaxis is recommended in all hospitalized patients with COVID-19 unless the risk of bleeding on prophylactic anticoagulation is higher than the risk of thrombosis. In non-hospitalized patients with COVID-19, pharmacological prevention of VTE is not recommended, unless the patient has other indications for the therapy or participates in a clinical trial [4,5]. Nevertheless, the incidence of arterial thrombosis (AT) in severe/critically ill COVID-19 patients admitted to intensive care units (ICU) across the five cohort studies was 4.4% (95% confidence interval [CI] 2.8–6.4) [6]. Thus, this raises a question about the need for antiplatelet therapy (APT) in these patients. In this article, we review the current knowledge about the use of APT and its efficacy and safety in COVID-19 patients.

## 2. COVID-19 and Arterial Thrombosis

The clinical manifestations of COVID-19 disease are variable, and the risk of AT seems to be dependent on the severity of the disease [7,8].

### 2.1. COVID-19 and the Incidence of Arterial Thrombosis

The incidence of VTE in COVID-19 patients ranged from 1.7 to 16.5% in 35 observational studies reported worldwide [9]. In a multicenter, cohort, retrospective database analysis of COVID-19 patients (with 3531 patients), the reported incidence of VTE was 6.68% [10]. However, reports describing the incidence of AT are inconsistent [11,12]. In a retrospective analysis of the RECOVER database, which enrolled 26,974 patients, the incidence of AT was 0.13% in COVID-19-positive patients. These patients experienced a greater proportion of AT in peripheral arteries [13]. In a retrospective U.S. cohort study, a comparison between a hospital stay due to COVID-19 and a hospital stay due to influenza showed a higher risk for venous, but not arterial, thrombotic events in the former [14]. On the other hand, in a recently published study analyzing 909,473 COVID-19 cases, AT ranged from 0.1% to 0.8% and increased to 3.1% among those who needed in-hospital admission. The occurrence of VTE and AT in patients with COVID-19 carried an increased risk of death (adjusted hazard ratios [HR] for VTE 4.42 [3.07–6.36] for those not hospitalized and 1.63 [1.39–1.90] for those hospitalized; and adjusted HR for AT 3.16 [2.65–3.75] and 1.93 [1.57–2.37], respectively) [15]. In summary, based on the latest available data, the incidence of AT in patients with COVID-19 is thought to be around 1% [13,14,15].

### 2.2. Pathophysiology of Arterial Thrombosis in COVID-19 Patients

COVID-19 is known to be associated with several abnormalities in hemostasis, including coagulopathy, activation of platelets, vessel injury, and alterations in fibrinolysis, which may be responsible for thrombosis related to this disease [1,16]. Such pathophysiological changes may cause AT or VTE, especially in patients with a severe course of the disease. These events occur more frequently in the lung, where both macro- and microthrombi have been reported [17]. In a post-mortem study, fibrin- and platelet-rich thrombi in pulmonary arterioles were reported together with congestion in capillaries and alveolar bleeding [18,19]. Subsequently, there is a greater chance of platelet aggregation and activation of the coagulation system due to COVID-19-related endothelial dysfunction. Furthermore, COVID-19 is related to platelet activation which has been repeatedly described in patients suffering from this disease [20,21,22]. Yatim et al. [20] reported that elevated soluble P-selectin (a marker of platelet activation) was associated with disease severity and in-hospital mortality and predicted the need for intubation and mechanical ventilatory support. Another observational, prospective study performed by Jakobs et al. on a sample of hospitalized patients with COVID-19 showed that adenosine diphosphate- (ADP), thrombin receptor activator peptide 6- (TRAP), and arachidonic acid- (AA) induced platelet reactivity was significantly higher in those with COVID-19 [22]. In addition, there is a dysregulation of the renin-angiotensin system due to SARS-CoV-2-induced consumption of the angiotensin-converting enzyme 2 (ACE-2) resulting in an intensive immune response that might lead to further endothelial damage [23], and aggravate the risk of AT development. There are several pathways of platelet-coagulation system activation with subsequent thrombus formation; therefore, it is challenging to make an optimal decision regarding the antithrombotic strategy in COVID-19 patients.

Additionally, the pro-thrombotic state in COVID-19 patients has been linked to the increased formation of neutrophil extracellular traps (NETs) [24]. Although the exact mechanisms and signaling pathways involved in neurophil/platelet interaction which leads to increased NETs formation are not fully understood, it is known that platelets can activate neutrophils to form NETs and that NETs themselves can be detected in thrombi, which points to a possible interaction between inflammatory cells (neutrophils), platelets, and thrombosis [25]. This interaction had also been described in COVID-19-related arterial thrombosis [26]. Interestingly, Petito et al. [27] observed in their prospective study on COVID-19-related thrombosis that NETs, but not platelet activation, correlated with disease activity and predicted thrombosis. However, it is not currently known whether the formation of NETs can be modified (reduced) by the administration of antiplatelet (or anticoagulant) therapy as there is no study dealing with this issue.

## 3. COVID-19 and the Response to Aspirin

Aspirin (Acetylsalicylic acid) is one of the most commonly used drugs worldwide [28] for its anti-platelet, analgesic, anti-inflammatory, and anti-pyretic effect. Aspirin exerts its major activity by inhibiting the cyclooxygenase enzyme (COX), which exists in two forms: COX-1 and COX-2 [29]. As a result, it inhibits the conversion of arachidonic acid into prostaglandins and thromboxane. Its activity expands to several other target structures leading to a number of anti-inflammatory and anti-thrombotic effects [28,29].

Endothelial inflammation and the exposure of von-Willebrand factor to sub-endothelial collagen in COVID-19 patients, in turn, precipitate thrombus formation manifesting as AT or VT [30].

A previous study suggested that aspirin could, in theory, offer protection against the severe form of COVID-19 infection [30]. This theory was examined in a retrospective study, which included 35,370 patients with and also without active aspirin prescriptions prior to becoming infected with SARS-CoV-2. Aspirin significantly reduced the risk of mortality in this study by 32%. After propensity score matching and confounding covariate adjustments, mortality decreased from 6.3 to 2.5% at 14 days and from 10.5 to 4.3% at 30 days in the propensity-matched cohorts [31]. Confounding covariate adjustments included age, gender, comorbidities, and the Care Assessment Needs [CAN] 1-year mortality score. In the RECOVERY trial [32], 14,892 patients were eligible for randomization to aspirin (7351 patients) or usual care alone (7541 patients). In this trial, 150 mg of aspirin did not reduce 28-day mortality, and among patients who were not receiving invasive mechanical ventilation at randomization, aspirin therapy did not reduce the probability of progression to the composite outcome of invasive mechanical ventilation or death. Aspirin therapy was associated with an increase in the rate of being discharged alive within 28 days, but the magnitude of the effect was small (1% absolute difference). Afterwards, Chow et al. published the results of a retrospective cohort study assessing COVID-19 patients, who received aspirin within the period of one week before admission involving the first day of hospital stay as well. After adjustment to sex, ethnicity, age, body mass index (BMI), comorbidities, and beta-blocker use, patients taking aspirin showed a reduced risk of intensive care unit (ICU) admission, mechanical ventilation, and in-hospital mortality [33]. Furthermore, a study carried out by Meizlish et al. analyzed the efficacy of aspirin in patients with SARS-CoV-2 infection. In this study, using multivariate analysis, patients taking aspirin had a lower cumulative incidence of in-hospital death [34]. A meta-analysis evaluating 12 retrospective studies in SARS-CoV-2 positive patients demonstrated a clear benefit of aspirin therapy in preventing a fatal course of COVID-19 disease [35].

Despite the fact that many studies have demonstrated the benefit of aspirin in SARS-CoV-2 patients, there is also a certain degree of evidence in the literature arguing against its use. In a recently released single-center, open-label, randomized controlled trial, 900 COVID-19 patients (with positive PCR), who needed in-hospital treatment were randomized to receive either atorvastatin 40 mg, aspirin 75 mg, or both (N = 225) added to standard care for 10 days or until discharge, whichever came first, or only standard therapy (N = 226). The primary endpoint was clinical deterioration to the level ≥ 6 of the WHO Ordinal Scale for Clinical Improvement. There was no difference in the primary endpoint across the study groups (*p* = 0.463); hence, aspirin treatment in this study did not prevent clinical deterioration [36]. Another randomized, double-blind, placebo-controlled phase two clinical trial in adult patients with adult respiratory distress syndrome assigned patients randomly at a 1:1 ratio to aspirin (75 mg) or placebo, for a maximum of 14 days. The primary endpoint was defined as the value of the oxygenation index (OI) on day seven. In this study, no significant difference in day 7 OI was found (aspirin group: 54.4 ± 26.8; vs. placebo group: 42.4 ± 25; mean difference, 12.0; 95% CI, -6.1 to 30.1; *p* = 0.19) [37]. Finally, in a meta-analysis of 34 studies, including randomized controlled trials (3 trials), prospective cohort studies (4 studies), or retrospective studies (27 studies) on associations between aspirin or other antiplatelet therapy administration and all-cause mortality in COVID-19 patients performed by Su et al. [38], aspirin showed no significant effect on all-cause mortality in randomized controlled trials, decreased all-cause mortality by 15% in prospective studies, and reduced all-cause mortality by 20% in retrospective studies.

In summary, the anti-platelet, anti-inflammatory, anti-pyretic, and analgesic effects of aspirin seem to be promising in COVID-19 patients; however, the results of available studies are still controversial (Table 1). More studies are needed to better define recommendations for aspirin treatment in COVID-19 patients.

## 4. COVID-19 and the Response on P2Y12 ADP Receptor Blockers

As previously mentioned, the increased risk of AT and VT observed during moderate-to-severe COVID-19 disease is associated with the increased morbidity and mortality of these patients [8,9]. In addition, patients with COVID-19 were demonstrated to require lower amounts of thrombin for the aggregation of platelets compared to healthy controls [39,40]. Furthermore, as mentioned, there is a study demonstrating ADP-, TRAP-, and AA-induced platelet hyper-reactivity in COVID-19 [22]. This (ADP-induced) platelet hyperreactivity could be, in theory, affected by treatment with P2Y12 ADP receptor blockers (ADPRB). However, there is limited information about the efficacy of P2Y12 ADPRB in patients with COVID-19 (Table 2). A small case-control study was performed enrolling five patients with severe respiratory failure as a result of SARS-CoV-2 infection. These patients required helmet continuous positive airway pressure (CPAP) and received a dose of 25 μg/kg/body weight of tirofiban as bolus infusion, followed by a continuous infusion of 0.15 μg/kg/body weight per minute for 48 h. Prior to the tirofiban infusion, patients received 250 mg of aspirin and 300 mg of clopidogrel. Both antiplatelet drugs continued at a dose of 75 mg daily for 30 days. All controls received a prophylactic or therapeutic dose of heparin, according to local standard operating procedures. Patients consistently experienced a mean (SD) reduction in A-a O2 gradient of −32.6 mmHg (61.9, *p* = 0.154), −52.4 mmHg (59.4, *p* = 0.016), and −151.1 mmHg (56.6, *p* = 0.011; *p* = 0.047 vs. controls) at 24, 48 h, and 7 days after treatment [41]. This study included a very limited number of patients (only five patients were enrolled), and this should definitely be taken into consideration when interpreting these results. An open-label, bayesian, adaptive randomized clinical trial was designed to evaluate the benefits and risks of adding a P2Y12 ADPRB (ticagrelor/clopidogrel) to anticoagulant treatment among non-critically ill patients hospitalized for COVID-19. In this trial, patients were randomized to a therapeutic dose of heparin and a P2Y12 ADPRB (N = 293 [ticagrelor = 63.2%/clopidogrel = 36.8%]) or a therapeutic dose of heparin only (usual care, N = 269) in a 1:1 ratio for 14 days or until hospital discharge. The composite primary outcome was organ support-free days evaluated on an ordinal scale that combined in-hospital death and the primary safety outcome was a major bleeding event within the first 28 days. The median number of organ support-free days was 21 days (interquartile range [IQR], 20–21 days) among patients in the P2Y12 ADPRB group and 21 days (IQR, 21–21 days) in the standard treatment group (adjusted OR, 0.83 [95% credible interval (CrI), 0.55–1.25]), and a major bleeding event occurred in six patients (2.0%) in the P2Y12 ADPRB group and in two patients (0.7%) in the control group; so no benefit of P2Y12 ADPRB was found in a group of non-critically ill COVID-19 patients with a higher risk of bleeding [42]. Likewise, in the REMAP-CAP trial (Randomized, Embedded, Multifactorial Adaptive Platform Trial), 1557 critically ill adult COVID-19 patients were enrolled and randomized to receive either open-label aspirin (N = 565), a P2Y12 ADPRB (N = 455), or no antiplatelet therapy (control group; N = 529). The median for organ support-free days was 7 (IQR, −1 to 16) in both antiplatelet and control groups (median-adjusted OR, 1.02 [95% CrI, 0.86–1.23]; 95.7% posterior probability of futility) and among survivors, the median for organ support-free days was 14 in both P2Y12 ADPRB group and control group [43]. Thus, no benefit of the addition of P2Y12 ADPRB (clopidogrel or ticagrelor) was found in non-critically ill and critically ill SARS-CoV-2 patients. Furthermore, in the COVD-PACT (Prevention of Arteriovenous Thrombotic Events in Critically-ill COVID-19 Patients Trial) trial [44], a multi-center randomized trial, 290 patients who required intensive care unit level of care for COVID-19 were randomly assigned to clopidogrel (300 mg orally once on the day of randomization, followed by 75 mg orally once daily on subsequent days of in-hospital stay) or no antiplatelet therapy in addition to the standard or full-dose anticoagulation. The primary efficacy end-point of the study was a composite of venous and arterial thrombosis and the primary safety end-point was a composite of fatal or life-threatening bleeding. In this randomized trial, there were no differences in the primary efficacy or safety end-points with clopidogrel versus no antiplatelet therapy. On the other hand, in a recently published international multicenter prospective registry, COVID-19 patients were treated with aspirin (oral or venous), clopidogrel, ticlopidine, prasugrel, and ticagrelor, either with single or dual antiplatelet therapy and were compared with patients without antiplatelet therapy. Patients who received antiplatelet therapy had a shorter duration of mechanical ventilation (8 ± 5 days vs. 11 ± 7 days, *p* = 0.01); and lower mortality (log-rank *p* < 0.01, RR 0.79, 95% CI 0.70 to 0.94) compared to patients who did not receive antiplatelet agents [45]. There are no available data about the use of cangrelor in COVID-19 patients. In summary, a limited number of published studies on the role of P2Y12 ADPRB in SARS-CoV-2 infection have shown controversial results. Therefore, further studies will be needed in future.

## 5. COVID-19 and the Response to Glycoprotein (GP) IIb/IIIa Inhibitors

Glycoprotein (GP) IIb/IIIa inhibitors (GPIIb/IIIaI) are potent, rapid, and selective blockers of platelet aggregation. These agents might, in theory, facilitate the dissolution of blood clots and prevent the formation of new clots in COVID-19 patients [46,47]. Additionally, patients with COVID-19 and acute ST-segment elevation myocardial infarction (STEMI) were shown to have higher rates of multivessel thrombosis, stent thrombosis, and higher modified thrombus grade post-first-device implantation with consequently a higher use of glycoprotein IIb/IIIa inhibitors and thrombus aspiration compared to COVID-19 negative patients [46]. There is little evidence about the benefit of the use of GPIIb/IIIaI in patients with COVID-19 (Table 3), which comes from a case describing a successful outcome of GPIIb/IIIaI administration in a patient with a severe form of COVID-19 viral pneumonia and non-ST-elevation myocardial infarction (NSTEMI) [47]; and from the above-mentioned study including five patients with a laboratory-confirmed SARS-CoV-2 infection, severe respiratory failure, who received tirofiban infusion (together with dual antiplatelet therapy) and who consistently experienced a reduction in blood gas oxygen gradient [41]. Although several cases showed a possible benefit of GPIIb/IIIaI in patients with COVID-19, right now there is no study examining the specificity of GPIIb/IIIaI use in COVID-19 versus non-COVID-19 cardiac patients. Furthermore, to date, there is no prospective, randomized trial to confirm this benefit, and further studies will be needed to adopt any final recommendations.

## 6. The Effect of Time of Antiplatelet Agent Admission on COVID-19-Related Outcomes

Another possible question is whether there is a difference between patients already on antiplatelet therapy before admission and those randomly assigned to antiplatelet drugs or placebo on top of anticoagulation after admission.

Looking at the studies in which antiplatelet therapy was started at the time of patient admission (patients who used antiplatelet therapy prior to admission were excluded) [32,34,36,37,43,44], only a single observational study performed by Meizlish et al. [34] showed a reduced risk of in-hospital death in patients receiving aspirin. In the rest of the studies, no overall benefit of antiplatelet therapy (either with aspirin or with a P2Y12 ADPRB) was shown.

In contrast, in studies which included patients with pre-event antiplatelet therapy [31,35,45], antiplatelet therapy reduced mortality [31], the risk of a fatal course of COVID-19 [35] and the risk of mortality or the duration of mechanical ventilation [45]. This observation could suggest that previous antiplatelet therapy could be more beneficial than starting the therapy after the estimation of the diagnosis of COVID-19, or that pre-existing antiplatelet therapy should not be stopped after the diagnosis of COVID-19. However, due to different study designs, it is difficult to compare their results, and these differences should be interpreted with caution until a study directly comparing pre-event antiplatelet therapy with an on-admission one is performed and published.

Summarizing, although the results of trials published so far indicate that antiplatelet agents might protect against the development of AT complications of severe COVID-19 disease, current recommendations [4,5] state that in non-hospitalized patients with symptomatic COVID-19, the initiation of antiplatelet therapy is not effective (does not reduce risk of hospitalization, arterial or venous thrombosis, or mortality) [48]. Among non-critically ill patients hospitalized for COVID-19, there is a strong recommendation against the addition of an antiplatelet agent. Adding an antiplatelet agent to prophylactic anticoagulation might be considered in selected critically ill patients (although the selection of patients is not well established). Nevertheless, only randomized studies with a minimum sample size of 100 patients and observational studies with a minimum sample size of 400 patients were included in the preparation of these recommendations [5]. As repeatedly discussed within the article, the majority of studies on antiplatelet agents had limited patient samples and therefore were not included in current recommendations. In addition, there is a report showing that the addition of an antiplatelet agent (aspirin) might be beneficial in frail cardiovascular patients, as was shown, for example, in the HOPE COVID-19 registry [49]. Going further, right now there is no satisfactory explanation for the differences between the results observed in the randomized and non-randomized clinical studies. One should consider the usual disadvantages of non-randomized and retrospective studies, such as selection bias or missing data; however, non-randomized studies might better copy the settings of real-world clinical practice and so far, available randomized trials also have their limitations (mostly relatively low sample sizes, exclusion criteria limiting the ability to enroll the patients in the highest risk, etc.). Therefore, the role of antiplatelet therapy in patients with COVID-19 disease should be probably re-questioned in future treatment recommendations.

## 7. Conclusions

The results of trials published so far indicate that antiplatelet agents (especially if administered prior to the development of the disease) might protect against AT complications of COVID-19; however, this evidence comes mostly from non-randomized studies and is not in line with current recommendations. Randomized controlled trials (with sufficient patient samples) are highly required to investigate whether pre-existing or newly added antiplatelet therapy might be beneficial in SARS-CoV-2 infection.

## Figures and Tables

**Table 1:** Aspirin therapy in patients with COVID-19 [31,32,33,34,35,36,37,38].

Author	Study Design	ICU Care	Number of Patients	Conclusion
Osborne T.F et al., 2021 [31]	Retrospective, cohort	Not reported	32,836	Aspirin was strongly associated with decreased mortality rates for Veterans with COVID-19.
RECOVERY Collaborative Group 2022, [32]	Randomised, open-label, platform trial	Not reported (non-invasive or invasive respiratory support in 33% of patients)	14,892	Aspirin did not reduce 28-day mortality, and in patients who were not on invasive ventilation at randomisation, aspirin did not reduce the probability of the composite outcome of mechanical ventilation or death
Chow J.H. et al. 2022, [33]	Observational, cohort	No (patients with moderate disease severity included)	112,269	Early aspirin use was associated with lower odds of 28-day in-hospital mortality.
Meizlish M.L. et al., 2021 [34]	Retrospective study	Not reported	2785	Aspirin therapy was associated with a lower incidence of in-hospital death
Kow C.S. et al., 2021 [35]	Meta-analysis of retrospective studies	Not reported	14,377	Significantly reduced risk of a fatal course of COVID-19 with the use of aspirin in patients with COVID-19.
Ghati N. et al., 2022 [36]	Randomized, open-label, controlled	Not reported	900	Aspirin treatment among patients admitted with mild to moderate COVID-19 infection did not prevent clinical deterioration
Toner P. et al., 2022 [37]	Randomized, placebo-controlled	Yes	49	Aspirin did not improve oxygen index or other physiological outcomes.
Su W. et al., 2022 [38]	Meta-analysis of randomized controlled, prospective cohort, and retrospective studies	Not reported	233,796	Aspirin reduced all-cause mortality in prospective and retrospective studies; no impact in randomized controlled studies

**Table 2:** P2Y12 ADPRB therapy in patients with COVID-19 [41,42,43,44,45].

Author	Study Design	ICU Care	Number of Patients	Conclusion
Veicca M. et al., 2020 [41]	Prospective, case series	Yes	5	Improvement in blood oxygenation with combined antiplatelet therapy (including P2Y12 ADPRB)
Berger J.S. et al., 2022 [42]	Open-label, bayesian, adaptive randomized clinical trial	No	562	P2Y12 ADPRB therapy did not result in an increased odds of improvement in organ support–free days during hospitalization
Bradbury C.A. et al., 2022 [43]	Prospective, adaptive platform trial	Yes	1824	P2Y12 ADPRB therapy did not result in an increased odds of improvement in organ support–free days during hospitalization
Bohula E. A. et al., 2022 [44]	Open-label, randomized, controlled trial	Yes	292	No effect of P2Y12 ADPRB on thrombotic complications
Santoro F. et al., 2022 [45]	Multicentre international prospective registry	No (only 9% of enrolled patients were admitted to ICU)	7824	Antiplatelet therapy (including P2Y12 ADPRB) was associated with lower mortality and shorter duration of mechanical ventilation

**Table 3:** GPIIb/IIIaI therapy in patients with COVID-19.

Author	Study Design	ICU Care	Number of Patients	Conclusion
Veicca M. et al., 2020 [41]	Prospective, case series	Yes	5	Improvement in blood oxygenation with combined antiplatelet therapy (including GPIIb/IIIaI)
Merrill P.J. and Bradburne R.M., 2021 [47]	Case report	Yes	1	Successful use of GPIIb/IIIaI for NSTEMI

## Data Availability

The source data are available at the Corresponding Author upon request.
